# Virome Diversity in Three Bat Species in Different Environments in Southern Spain

**DOI:** 10.3390/microorganisms14092025

**Published:** 2026-09-11

**Authors:** Aline Méndez-Rodríguez, Juan Ledesma, Celia López-González, Heliot Zarza, Ricardo López-Wilchis, Nuria Labiod, Isabel Cuesta, Inmaculada Casas, Juan E. Echevarría, Ana Vázquez, Javier Juste

**Affiliations:** 1 Doctorado en Ciencias Biológicas y de la Salud, Universidad Autónoma Metropolitana, Ciudad de Mexico 14387, Mexico; aline_mera@hotmail.com; 2 Unidad de Bioinformática, Unidades Centrales Científico Técnicas, Instituto de Salud Carlos III, 28029 Madrid, Spain; juan.ledesma@isciii.es (J.L.); isabel.cuesta@isciii.es (I.C.); 3 Instituto Politécnico Nacional, Centro Interdisciplinario de Investigación para el Desarrollo Integral Regional (CIIDIR) Unidad Durango, Sigma 119 Fraccionamiento 20 de Noviembre II Durango, Durango 34220, Mexico; celialg.dgo@gmail.com; 4 Departamento de Ciencias Ambientales, Ciencias Biológicas de la Salud, Universidad Autónoma Metropolitana-Lerma, Av. de las Garzas 10, Col. El Panteón, Lerma 52005, Mexico; h.zarza@correo.ler.uam.mx; 5 Departamento de Biología, Universidad Autónoma Metropolitana-Iztapalapa, Av. San Rafael Atlixco 186, Col. Vicentina, Iztapalapa 09340, Mexico; rlw@xanum.uam.mx; 6 Centro Nacional de Microbiología, Instituto de Salud Carlos III, 28029 Madrid, Spain; nlabiod@isciii.es (N.L.); icasas@isciii.es (I.C.); jeecheva@isciii.es (J.E.E.); 7 Centro de Investigación Biomédica en Red de Enfermedades Infecciosas, Centro de Investigación Biomédica en Red, 28029 Madrid, Spain; 8 Centro de Investigación Biomédica en Red de Epidemiología y Salud Pública, 28220 Madrid, Spain; 9 Estación Biológica de Doñana, Consejo Superior de Investigaciones Científicas, Avda. Américo Vespucio 26, 41092 Sevilla, Spain

**Keywords:** *Cnephaeus isabellinus*, *Eptesicus*, metagenomics, *Nyctalus lasiopterus*, *Pipistrellus kuhlii*, Vespertilionidae, virology

## Abstract

Viruses represent a major component of global biodiversity and are integral to ecological and evolutionary processes shaping host populations and communities. Bats, with high species richness and ecological diversity, provide a system to examine how host traits and environmental context structure viral communities. Here, we characterize viral diversity from three bat species (*Pipistrellus kuhlii*, *Cnephaeus isabellinus*, and *Nyctalus lasiopterus*) using samples across two environments in southern Spain: a well-preserved Mediterranean forest (Sierras de Cazorla, Segura, and Las Villas Natural Park, CSVNP) and a human-modified wetland-agrosystem mosaic (Doñana National Park, DNP). Metagenomics detected 72 eukaryotic virus species, including viruses reported in mammals, insects, arachnids, and plants. Viral richness and composition varied among samples, bat species, and environments. Samples of *P. kuhlii* exhibited the highest richness, driven by insect-associated viruses. *C. isabellinus* showed a higher contribution of vertebrate-related viruses, whereas *N. lasiopterus* exhibited the lowest richness. CSVNP samples showed higher viral richness and more exclusive taxa, whereas DNP samples exhibited lower richness, with a greater contribution of arthropod-associated viruses, potentially reflecting prey communities. Despite this, diversity metrics were similar between environments, indicating structurally comparable communities composed of distinct taxa. Bat viromes appear to be associated with host ecology, trophic behavior, and environmental context.

## 1. Introduction

Viruses are the most diverse and abundant biological entities on Earth and are found in nearly all organisms and environments, with the possible exception of certain endosymbiotic bacteria [1]. Beyond their ubiquity, viruses play a fundamental ecological and evolutionary role, shaping host evolution through gene transfer and innovation, as well as modulating population dynamics and community structure [2,3]. At the same time, viruses are of major public health concern because some can cross species barriers, causing diseases and, in some cases, death in new hosts [4].

Historically, virological research has focused on viruses associated with plants and animals that are directly relevant to humans [4]. In recent times, this perspective has expanded substantially with the development of metagenomic next-generation sequencing (mNGS) technologies, which enable a more comprehensive characterization of viral diversity [4]. Unlike the traditional Polymerase Chain Reaction (PCR) approach, which generally targets known or closely related viruses, mNGS enables the detection of a much broader range of viral entities, including previously overlooked viruses, thereby improving our understanding of the entire virome and its interactions with their hosts [5].

Despite the growing technological advances, our understanding of viruses present in animals remains incomplete and strongly biased toward a limited number of host taxa [6,7]. As a result, vertebrate groups such as rodents, birds, and bats have received considerably more attention than most other wildlife taxa [8,9]. This reflects not only the association of some species with zoonotic viruses, but also their ecological plasticity, abundance, and broad geographic distribution across diverse ecosystems [8]. Rodents harbor a wide range of viruses, including hantaviruses, arenaviruses, and orthopoxviruses [10], while birds harbor globally circulating viruses, including avian influenza viruses and multiple arboviruses [11]. Bats (Chiroptera: Mammalia) in particular have been recognized as reservoirs of an exceptionally diverse assemblage of viruses [12]. To date, viruses from at least 33 families have been identified in bats, including several associated with zoonotic disease outbreaks, such as Hendra and Nipah paramyxoviruses, Ebola and Marburg filoviruses, and coronaviruses that are evolutionarily related to the recent Severe Acute Respiratory Syndrome Coronavirus 1 (SARS-CoV-1), Severe Acute Respiratory Syndrome Coronavirus 2 (SARS-CoV-2), and Middle East Respiratory Syndrome Coronavirus (MERS-CoV) disease outbreaks [13,14].

The viral diversity observed in bats has been linked to evolutionary and ecological traits distinctive of this group [12]. These include flight and long-distance movement, which facilitates virus dispersal; exceptional longevity, which allows prolonged virus persistence; the gregarious behavior of many species, with colonies ranging from a few individuals to millions, which increases contact rates; and immune responses capable of controlling viral replication with limited inflammation [12,15,16,17]. Together, these traits may enable bats to harbor particularly diverse viral communities while rarely exhibiting clinical signs of disease, making them valuable systems for understanding long-term host–virus associations and the ecological drivers of virome composition [12,15,16,17].

However, the remarkable viral richness documented in bats is not uniformly distributed among species [18,19]. Considerable variation in both virus richness and virome composition has been reported among bat taxa [18,20,21]. This pattern suggests that, beyond the general traits shared by Chiroptera, taxon-specific ecological and evolutionary characteristics also contribute to shaping viral communities. Understanding the factors underlying this variation remains a major challenge in bat virology and disease ecology.

Current evidence suggests that bat viromes are influenced by multiple interacting factors that remain incompletely understood. Bat host ecological traits that were reported to influence viral diversity significantly or virus richness among bat species include diet [22,23], movement patterns, demographics [24], mating strategies [24,25], litter size, and longevity [8]. In addition to sex [22,26], age [27,28], phylogenetic diversity [29], and population genetic structure [30].

While host biological characteristics are important determinants of viral diversity, host–virus interactions also occur within ecological systems that are increasingly modified by human activities [27]. Over recent decades, deforestation, habitat fragmentation, urban expansion, agricultural intensification, and livestock farming have transformed natural ecosystems worldwide, altering habitat structure, species abundance, and distribution patterns [31,32,33]. These environmental changes can modify ecological interactions within wildlife communities, including those between hosts and viruses, by modifying contact networks, resource availability, and community composition [34]. Consequently, whether biodiversity loss amplifies or dilutes pathogen transmission remains an active area of debate [35,36,37,38].

Bat viromes likely reflect the combined influence of host biology and environmental conditions. Consequently, bat viral diversity is expected to reflect not only environmental conditions but also the ecological changes induced by habitat modification [36]. Comparative studies that evaluate multiple host species across contrasting environments provide a valuable framework for identifying the mechanisms shaping virome composition.

In this context, this study uses metagenomic sequencing to analyze viral diversity in samples from three closely related vespertilionid bat species with contrasting ecological characteristics: the small, sedentary, generalist insectivore *Pipistrellus kuhlii*; the medium-sized, predominantly sedentary insectivore *Cnephaeus isabellinus*; and the large, seasonally migrant, *Nyctalus lasiopterus*, which is primarily insectivorous but exhibits a unique trophic strategy that includes seasonal predation on migratory birds [39,40]. These differences in movement patterns, habitat use, and diet provide an opportunity to examine how host ecological traits may be associated with viral diversity.

The three species were sampled in two contrasting environments in southern Spain: the relatively well-preserved Mediterranean forests of the Sierras de Cazorla, Segura, and Las Villas Natural Park (CSVNP) and the human-modified wetland-agrosystem mosaic of Doñana National Park (DNP).

By integrating host ecological traits in an environmental context, this study aims to examine how host ecology and environmental conditions are associated with viral diversity across bat species and habitats. Based on the ecological and evolutionary framework outlined above, we hypothesized that viral diversity would differ among bat species according to their ecological traits and would be higher in the environmentally modified landscape due to altered ecological interactions and increased opportunities for cross-species viral exchange. Overall, this work will contribute to a broader understanding of how viromes are structured in wildlife across conserved and modified landscapes, highlighting the role of interactions among habitat structure, host assemblages, and trophic pathways.

## 2. Materials and Methods

### 2.1. Sample Areas and Sample Collection

**Figure 1:**
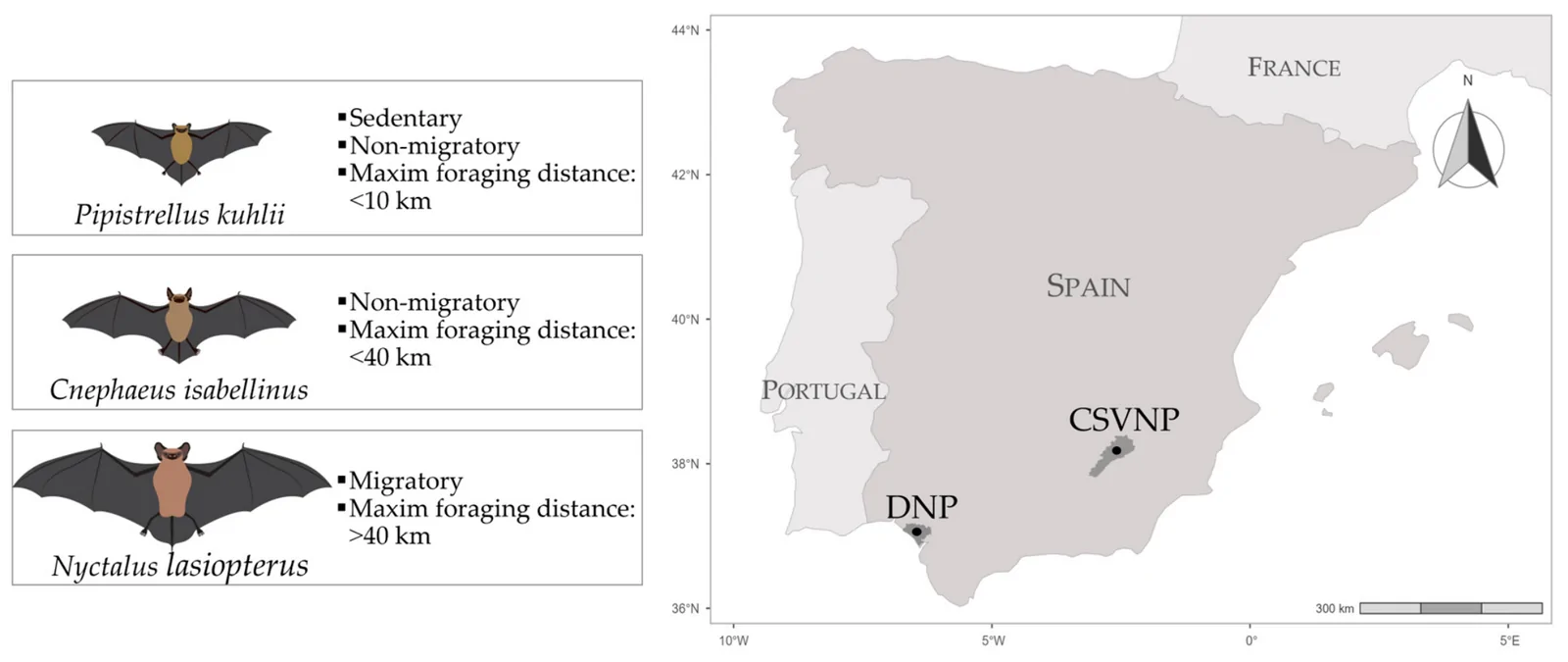
Ecological characteristics of the bat species analyzed in this study (*Pipistrellus kuhlii*, *Cnephaeus isabellinus*, and *Nyctalus lasiopterus*) and geographic location of the study sites southern Spain: Sierras de Cazorla, Segura, and Las Villas Natural Park (CSVNP) (approximately 38°52′ N, 2°15′ W and 37°35′ N, 3°16′ W), and Doñana National Park (DNP) (37°07′ N, 6°12′ W and 36°40′ N, 6°34′ W), where samples were collected.

The three bat species were sampled in two contrasting environments in southern Spain: the Sierras de Cazorla, Segura, and Las Villas Natural Park (CSVNP) and Doñana National Park (DNP) (Figure 1). The two study areas are located several hundred kilometers apart in the southern half of the Iberian Peninsula.

The CSVNP represents a relatively well-preserved large ecosystem covering more than 200,000 ha and dominated by coniferous and oak forests. The region is characterized by complex topography and pronounced altitudinal gradients, which generate a wide variety of microhabitats and local climatic conditions [41,42]. These environmental characteristics provide a high degree of diversity in potential roosting sites for bats. The park hosts nearly 70% of all bat species recorded in the Iberian Peninsula [41,43].

In contrast, DNP is a large ecosystem covering more than 70,000 ha and comprising a mosaic of habitats surrounding the largest wetland in Western Europe. Although recognized as a biodiversity hotspot, it hosts fewer bat species [13,14]. The landscape has been extensively modified by agriculture, livestock farming, and groundwater extraction, generating a heterogeneous mosaic of natural and human-altered habitats [13,44,45]. The area is characterized by marshes and grasslands near the mouth of the Guadalquivir River.

A total of 32 individuals of *P. kuhlii*, 20 of *C. isabellinus*, and 26 of *N. lasiopterus* were captured using mist nets placed near water bodies between 19:00 and 00:00 h at the two study environments in Spain: CSVNP and DNP (Table 1). Fieldwork was conducted in the Spring, between May–July 2023 and May 2024, under collection permits granted by the Spanish Consejería de Agricultura, Ganadería, Pesca y Desarrollo Sostenible, Dirección General de la Producción Agrícola y Ganadera. Species were easily distinguished by differences in body size and confirmed using the keys by Dietz and Von Helversen [46]. Data on external measurements, age, and sex of the specimens were recorded before the animals were released at the same capture site.

For virome characterization, saliva and/or fecal samples were collected. Saliva was obtained with oropharyngeal swabs. To collect fecal material, bats were kept in cloth bags for 30 min, after which fecal samples were recovered from the bags or directly from the bat’s body using sterile tweezers.

Samples were pooled into groups of up to five bats, with each pool containing individuals that shared the same sample type (saliva or fecal), bat species, and collection environment (location) (Table S1). Pooling samples allowed multiple individuals to be processed within a single library but resulted in a loss of individual-level resolution, preventing the assessment of viral prevalence. Each pooled sample was stored in an Eppendorf tube containing 1 mL of Minimum Essential Medium (MEM) and kept at 4 °C during fieldwork and at −80 °C in the laboratory until processing.

### 2.2. Nucleic Acid Extraction, Library Preparation, and Sequencing

Before nucleic acid extraction, saliva and fecal samples underwent treatment to remove as much eukaryotic cellular genetic material as possible. This process involved vortexing the samples at maximum speed for 2 min, followed by centrifugation at 17,000× *g* for 3 min. The supernatant was then subjected to sequential filtration using Ultrafree-MC-SV Centrifugal Filters Durapore PVDF (Merck KGaA, Darmstadt, Germany) with 5.0 µm and 0.45 µm pore sizes, with centrifugations at 12,000× *g* for 3 min at each step.

Additionally, saliva samples underwent nuclease treatment to reduce host-derived, unencapsulated nucleic acids prior to extraction. This additional treatment was applied only to saliva samples because it was implemented specifically to address the higher host nucleic acid content observed in this sample type during protocol optimization. A total of 130 µL of the filtrate was mixed with 2 µL of benzonase nuclease, Sigma-Aldrich (St. Louis, MO, USA), 1 µL of micrococcal nuclease, New England Biolabs, and 7 µL of buffer (1 M Tris, 100 mM CaCl_2_, and 30 mM MgCl_2_, pH 8), and incubated at 37 °C for 2 h. The reaction was subsequently inactivated with 7 µL of 10 mM EDTA. After pretreatment, nucleic acids were extracted from 140 µL of the sample using the Viral RNA Mini Kit QIAGEN (QIAGEN, Hilden, Germany), according to the manufacturer’s instructions, with a final double elution in 30 µL.

Library preparation was performed using the NEBNext Ultra II Directional RNA Library Prep Kit for Illumina, New England Biolabs (Ipswich, MA, USA), following the manufacturer’s instructions without modifications. This protocol includes RNA fragmentation and random priming followed by first- and second-strand cDNA synthesis and library construction. Because DNA was not specifically removed from the nucleic acid extracts, DNA molecules present in the extracts could also be retained during library preparation, allowing the detection of DNA viruses in addition to RNA viruses. Purification was conducted with NEBNext AMPure beads from Beckman Coulter. In addition to the sample libraries, a distilled water blank library was included as a negative control. Libraries were quantified using a Quantus fluorometer (Promega, Madison, WI, USA), and fragment lengths were verified using an Agilent 2100 Bioanalyzer (Agilent Technologies, Santa Clara, CA, USA). Finally, sequencing was performed on a NovaSeq 6000 (Illumina, San Diego, CA, USA) using the SP Reagent v1.5 kit (Illumina, San Diego, CA, USA), generating 150 bp paired-end reads.

### 2.3. Bioinformatics Data Analysis

NGS data were analyzed for viral genome reconstruction using viral pipeline v2.6.0 (https://github.com/nf-core/viralrecon, accessed on 31 October 2024), written in Nextflow (https://www.nextflow.io/) in collaboration between the nf-core community (https://nf-co.re/) and the Bioinformatics Unit at the Carlos III Health Institute (BU-ISCIII). Briefly, fastq files were first quality-assessed using FastQC v0.11.9 [45]. Then, they were trimmed. Then, the trimmed reads were quality assessed using Fastp v.0.23.2 [46]. Resulting reads were filtered to remove host sequences using Kraken 2.1.2 [47] with a database composed of the genomes of *Pipistrellus pipistrellus* (GCA_903992545.1), *P. kuhlii* (GCF_014108245.1), *Antrozous pallidus* (GCA_027563665.1), *Eptesicus fuscus* (GCF_027574615.1), and *Myotis brandtii* (GCF_000412655.1). *De novo* assembly was performed using SPAdes 3.15.5 [48], and viral sequences from the contigs were identified using Blast 2.11 and the viral nucleotide GenBank NCBI database (release 248) [49].

Viral contigs identified as bacteriophages and those covering less than 300 nucleotides of a reference genome were excluded. For contigs with multiple hits, the best bit score was used to select a unique hit, which was then used to determine the accession number of the closest viral reference genome. These reference genomes were subsequently used for mapping with Bowtie2 v2.4.4 [50]. Mapping statistics were generated using Picard v3.0.0 (https://github.com/broadinstitute/picard, accessed on 31 October 2024) and SAMtools v1.16.1 [51]. Variant calling was performed using iVar variants v1.4 [52]. Viral genome consensus sequences were generated using BEDtools v2.30.0 [53], considering variants with an allele frequency greater than 80% and masking genomic regions with coverage lower than 10 reads. Only genomes with at least 100 reads mapped to a given reference genome were considered for further analyses. Finally, sequences belonging to the same viral family were identified and compared through sequence alignment to assess their level of similarity. This sequential approach combined contig-level sequence similarity and read-level confirmation against the corresponding viral reference genome with stringent filtering criteria to improve the reliability of viral identification. Finally, for taxonomic assignment, we used the level of similarity between our sequences and known viral sequences through BLAST. We retained viral sequences with≥95% nucleotide identity to the corresponding reference sequence as viral assignments, whereas sequences with lower nucleotide identity were considered putative viral taxa.

To summarize, we use a sequential approach combining contig-level sequence similarity and read-level confirmation against the corresponding viral reference genome, together with stringent filtering criteria, to maximize the reliability of viral identification.

### 2.4. Viral Diversity Comparisons

We described and compared viral diversity patterns for each bat species and between sampling localities. Viral diversity was estimated at two taxonomic levels: (1) viral species and (2) viral families. For all analyses, read counts were normalized as reads per million (RPM).

We calculated viral richness (S), evenness index (E), and Shannon diversity (H′) by bat species and study environment. Differences in Shannon diversity between localities for each bat species were assessed using the Shantest_C function in MATLAB, which was modified from the original shantest function developed by Christopher Higgins (Arizona State University, Tempe, AZ, USA) and implemented in MATLAB R2020a Update 8 (9.8.0.1873465; The MathWorks, Inc., Natick, MA, USA).

Complementary to conventional diversity metrics, we quantified diversity using Hill numbers, or “true diversities” (^q^D) [54,55], considering values of q = 0, 0.25, 0.5, 1, 2, 4, and 8. Within this framework, ^0^D corresponds to species (or family) richness, depending on the taxonomic level analyzed; ^1^D is equal to the exponential of Shannon’s diversity index; and ^2^D represents the multiplicative inverse of Simpson’s diversity index [54]. Intermediate q-values describe how diversity changes as the weight given to dominant taxa increases, enabling comparisons across gradients of sensitivity to rare versus abundant viruses. Analyses were conducted in R version 2025.09.0+387 using the vegan package version 2.8-0 [53] with the renyi function.

## 3. Results

A total of 78 bat samples were collected and pooled into 36 libraries according to sample type, bat species, and sampling environment. After sequencing and quality filtering, 21 libraries met all criteria (see Materials and Methods) and were retained for subsequent analyses. These comprised eight pools of *P. kuhlii* (four from CSVNP and four from DNP), eight pools of *C. isabellinus* (five from CSVNP and three from DNP), and five pools of *N. lasiopterus* (four from CSVNP and one from DNP). Sequencing of these 21 libraries generated a total of 2,349,723,463 raw reads, of which 8,592,931 were classified as eukaryotic viral after quality trimming, filtering, and taxonomic assignment (Table 2). Because only one informative pool was obtained for *N. lasiopterus* in DNP, any observations regarding this species for this environment will be interpreted with caution. The number of pools for the other environment (CSVNP) was in the range of the other species instead (Table 2), and therefore this species/environment association was kept for further comparisons. No viral reads were detected in the negative control libraries after applying the same filtering and taxonomic assignment criteria used for the sample libraries.

**Figure 2:**
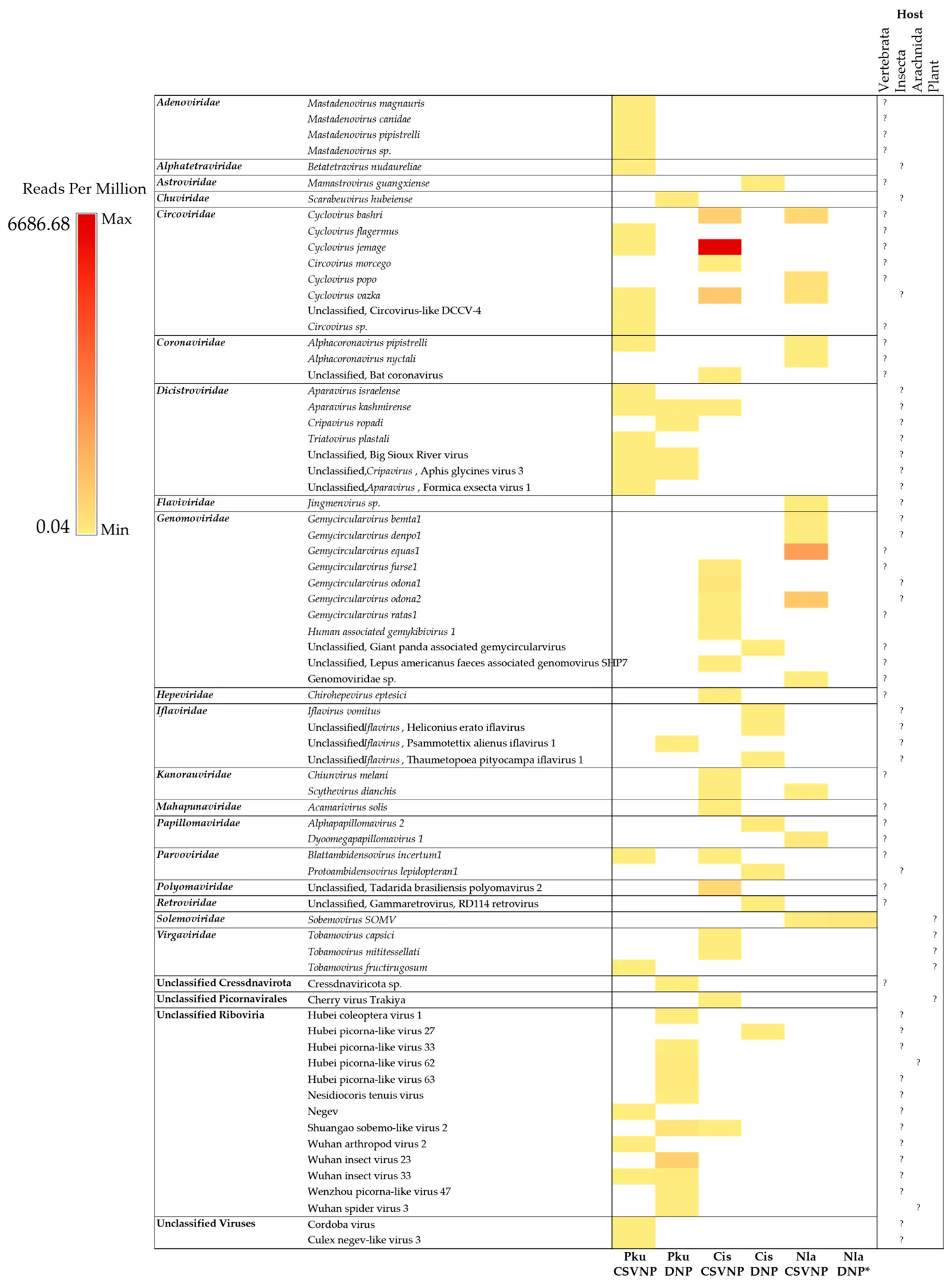
Heatmap of viral relative abundance (Reads Per Million, RPM) across combinations of bat species and environmental conditions. Color intensity indicates RPM abundance, whereas White cells indicate that no viral reads were detected in the corresponding sample. Both classified and unclassified viral taxa are included, together with their known hosts. Virus assignments showing ≥95% nucleotide identity to the corresponding reference sequence are indicated in bold. Acronyms represent bat species and sampling environments as follows: **PkuCSVNP** (*P. kuhlii* from CSVNP); **PkuDNP** (*P. kuhlii* from DNP); **CisCSVNP** (*C. isabellinus* from CSVNP); **CisDNP** (*C. isabellinus* from DNP); **NlaCSVNP** (*N. lasiopterus* from CSVNP); and **NlaDNP** (*N. lasiopterus* from DNP). An asterisk (*) indicates that the corresponding dataset was represented by a single library. ? = Uncertain host.

In total, 72 different putative viral taxa were detected. Out of these, 26 (36.1%) showed ≥95% nucleotide identity to a reference sequence and were considered as identified viruses, whereas the remaining 46 (63.9%) were maintained as putative viral taxa (Table S2). Out of the 72 putative viruses detected, 55 were assigned to 19 viral families, while the remaining 17 had no known taxonomic assignment (Figure 2). These taxonomically unclassified viruses have been previously reported in a wide range of hosts and samples, including plants (e.g., *Prunus avium*), arachnids (*Argiope bruennichi*), insects (e.g., beetles, odonates, hemipterans, and several mosquito species), and fecal samples of insectivorous vertebrates.

Among the 19 viral families identified, eight corresponded to Deoxyribonucleic Acid (DNA) viruses and eleven to Ribonucleic Acid (RNA) viruses, encompassing hosts ranging from vertebrates to plants. In viral species counts, 45.83% were related to insects, followed by vertebrates (40.28%), plants (6.94%), and arachnids (2.78%). The remaining 4.17% corresponded to viruses previously detected in environmental samples (Table S2). These host associations of the detected viral taxa were previously reported in the literature and should not be interpreted as evidence of infection or host association with bats.

The distribution of viral families varied across host species and habitats. Most viruses (81.90%) were restricted to a specific species-habitat combination, whereas only 18.10% exhibited a broader distribution across hosts or environments.

### 3.1. Viral Diversity

#### 3.1.1. Vertebrate Viruses

Twenty-nine vertebrate-related viral taxa were identified, accounting for 6,670,423 reads and distributed across 12 families, including *Adenoviridae*, *Astroviridae*, *Circoviridae*, *Coronaviridae*, *Genomoviridae*, *Hepeviridae*, *Kanorauviridae*, *Mahapunaviridae*, *Papillomaviridae*, *Parvoviridae*, *Polyomaviridae*, and *Retroviridae*, in addition to one unclassified species (Figure 2).

Some viral families were restricted to specific hosts or environments (e.g., *Adenoviridae* were detected exclusively in *P. kuhlii* from CSVNP, or *Astroviridae* and *Retroviridae* were detected only in *C. isabellinus* from DNP), whereas other viruses were shared across bat species within the same habitat (e.g., *Circoviridae* and *Coronaviridae* were detected in all bat species from CSVNP).

**Figure 3:**
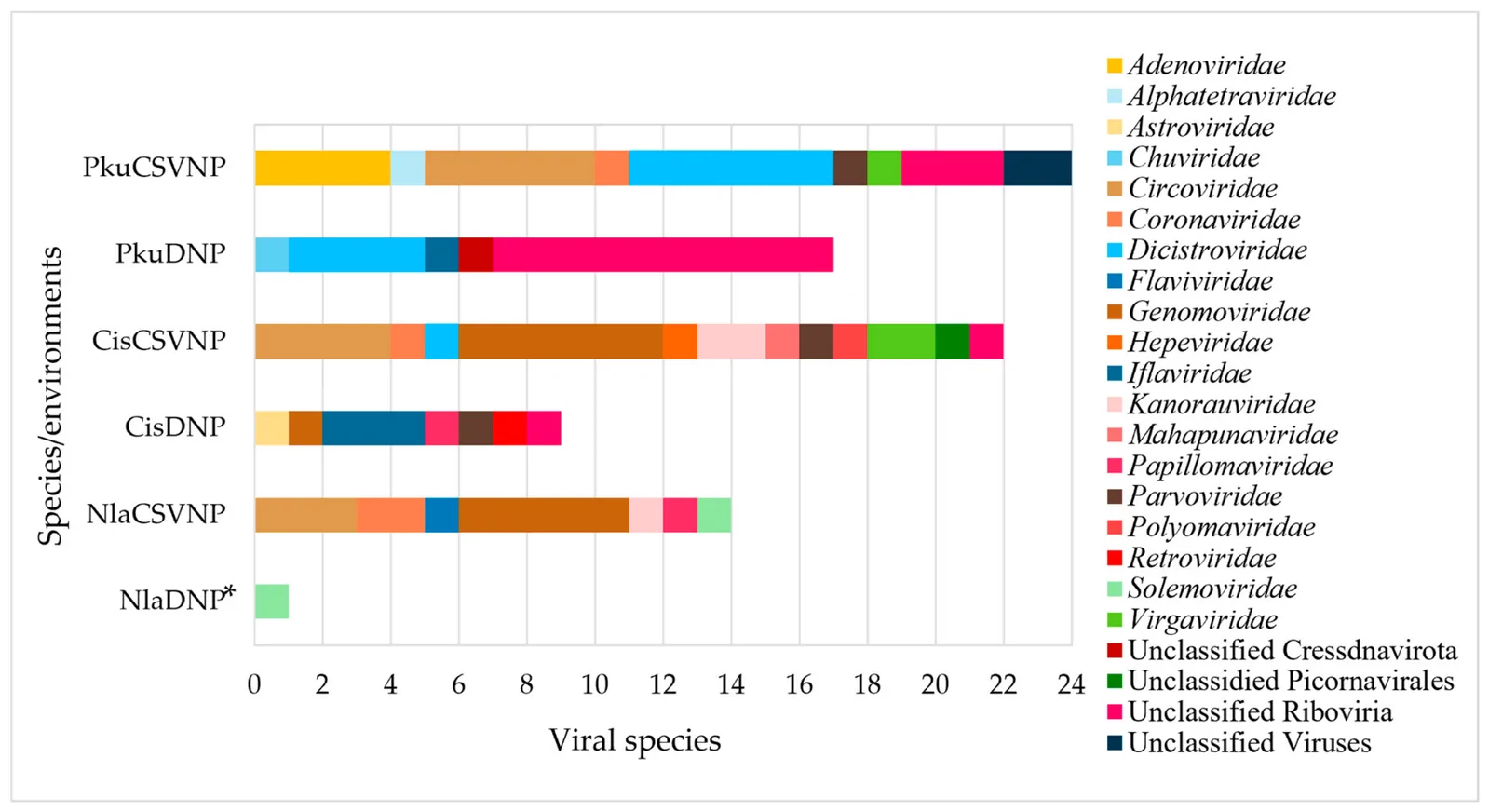
Number of viral taxa detected per viral family in samples from *P. kuhlii*, *C. isabellinus*, and *N. lasiopterus* in the two studied environments: CSVNP and DNP. Acronyms represent bat species and sampling environments as follows: **PkuCSVNP** (*P. kuhlii* from CSVNP); **PkuDNP** (*P. kuhlii* from DNP); **CisCSVNP** (*C. isabellinus* from CSVNP); **CisDNP** (*C. isabellinus* from DNP); **NlaCSVNP** (*N. lasiopterus* from CSVNP); and **NlaDNP** (*N. lasiopterus* from DNP). An asterisk (*) indicates that the corresponding dataset was represented by a single library.

Among vertebrate-related viruses, *Cyclovirus jemage* (*Circoviridae*) was the most abundant, occurring in *P. kuhlii* and *C. isabellinus* from CSVNP (Figure 3). Another vertebrate-related virus detected in *P. kuhlii* and *C. isabellinus* from CSVNP was *Blattambidensovirus incertum1* (*Parvoviridae*), previously reported in birds.

#### 3.1.2. Insect Viruses

A total of 33 insect-related viral taxa were detected. Of these, 20 species were classified into eight families: *Alphatetraviridae*, *Chuviridae*, *Circoviridae*, *Dicistroviridae*, *Flaviviridae*, *Genomoviridae*, *Iflaviridae*, and *Parvoviridae*, whereas the remaining 13 species lacked taxonomic classification. Collectively, these viruses represented 1,452,747 reads (Figure 2).

These viruses were associated with insects from Odonata, Hemiptera, Lepidoptera, Hymenoptera, Coleoptera, and Diptera. Some families were exclusive to a single host or environment (e.g., *Alphatetraviridae* in *P. kuhlii* from CSVNP, *Chuviridae* in *P. kuhlii* from DNP, and *Flaviviridae* in *N. lasiopterus* from CSVNP). In contrast, Dicistroviridae, *Genomoviridae*, *Iflaviridae*, and *Parvoviridae* occurred across multiple hosts or environments. *Cyclovirus vaska* (*Circoviridae*) was the most abundant and was detected in all three bat species from CSVNP (Figure 3).

#### 3.1.3. Arachnid Viruses

Two unclassified spider-related viruses (*Hubei picorna-like virus 62* and *Wuhan spider virus 3*) were detected exclusively in *P. kuhlii* from DNP, with a total of 6846 reads (Figure 2).

#### 3.1.4. Plant Viruses

Five plant-related viral taxa were detected, belonging to the families *Solemoviridae* and *Virgaviridae*, and to an unclassified group. These included *Sobemovirus SOMV*, three *Tobamovirus* species (*T. capsici*, *T. mititessellati*, and *T. fructirugosum*), and *Cherry virus Trakiya*, for a total of 4692 reads (Figure 2).

*Sobemovirus SOMV* was detected exclusively in *N. lasiopterus* from both environments, whereas *Virgaviridae* species were restricted to CSVNP: *Tobamovirus capsici*, *T. mititessellati*, and Cherry virus Trakiya in *C. isabellinus*, and *Tobamovirus fructirugosum* in *P. kuhlii*. These viruses have been reported in a diverse range of wild and cultivated plants, including several of agricultural and medicinal importance (Amaranthaceae, Apocynaceae, Balsaminaceae, Brassicaceae, Fabaceae, Nicotianoideae, Phrymaceae, Rosaceae, Saniculoideae, Solanaceae, and Solanoideae).

### 3.2. Comparative Analysis of Viral Diversity

**Figure 4:**
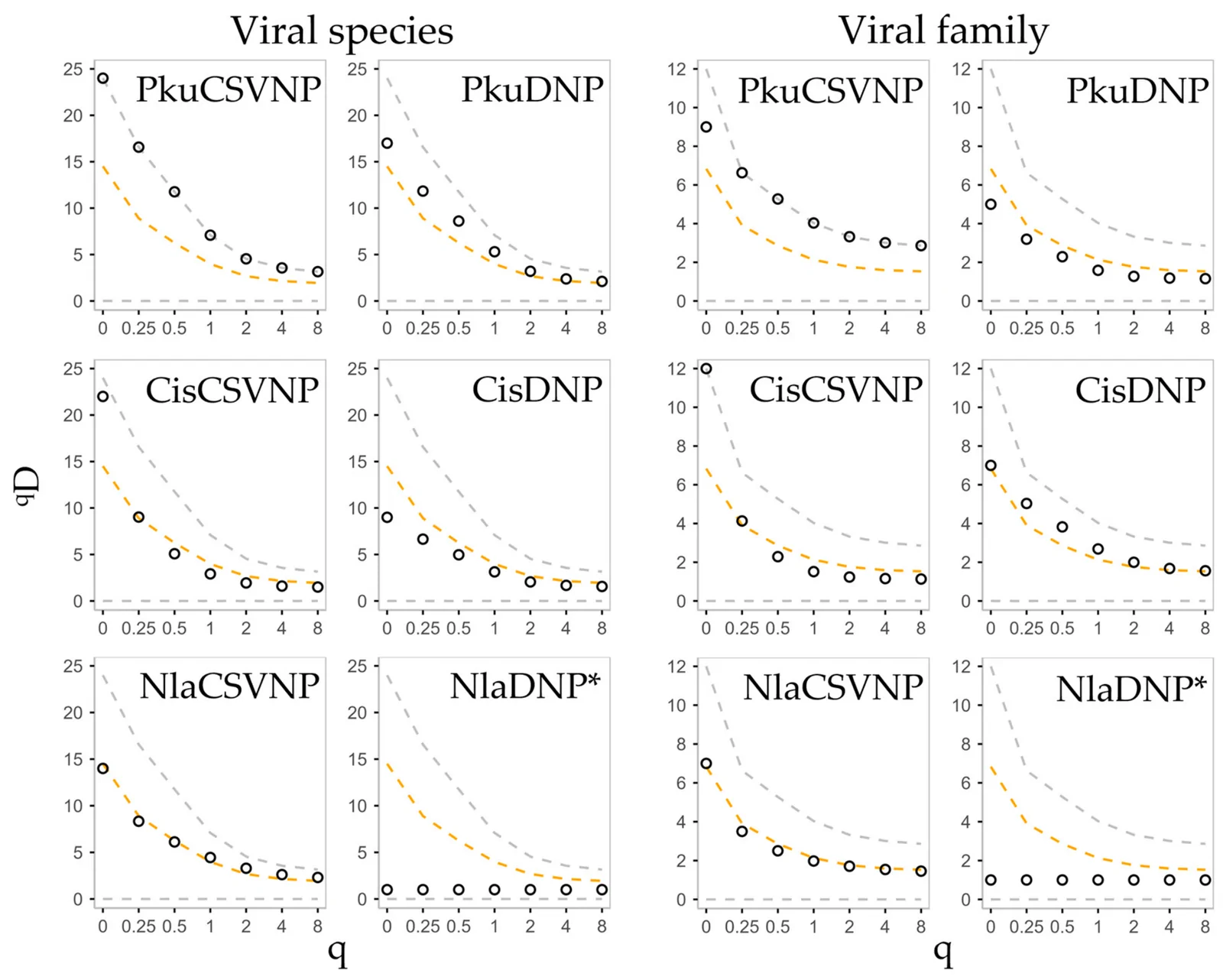
Diversity profiles based on Hill numbers (q = 0, 0.25, 0.5, 1, 2, 4, and 8) calculated from the relative abundance (RPM) of viral species and families detected for each bat species–environment combination. Circles represent individual Hill number estimates, the orange line indicates the mean Hill diversity trend, and gray lines denote the confidence intervals around the mean. Acronyms represent bat species and sampling environments as follows: **PkuCSVNP** (*P. kuhlii* from CSVNP); **PkuDNP** (*P. kuhlii* from DNP); **CisCSVNP** (*C. isabellinus* from CSVNP); **CisDNP** (*C. isabellinus* from DNP); **NlaCSVNP** (*N. lasiopterus* from CSVNP); and **NlaDNP** (*N. lasiopterus* from DNP). An asterisk (*) indicates that the corresponding dataset was represented by a single library.

A total of 72 viral taxa belonging to 19 families were detected in the study. Among them, the families *Circoviridae* and *Coronaviridae* were found in the three bat species. Viral diversity in the samples from each bat species was assessed using the Evenness index, Shannon diversity, and Hill numbers, allowing characterization of patterns of richness, evenness, and dominance in viral communities and comparison between hosts and environments at two taxonomic scales (viral species and families). Patterns of viral diversity were consistent across taxonomic levels, as the analyses based on viral families showed trends similar to those observed at the viral species level (Table 3; Figure 4).

**Figure 5:**
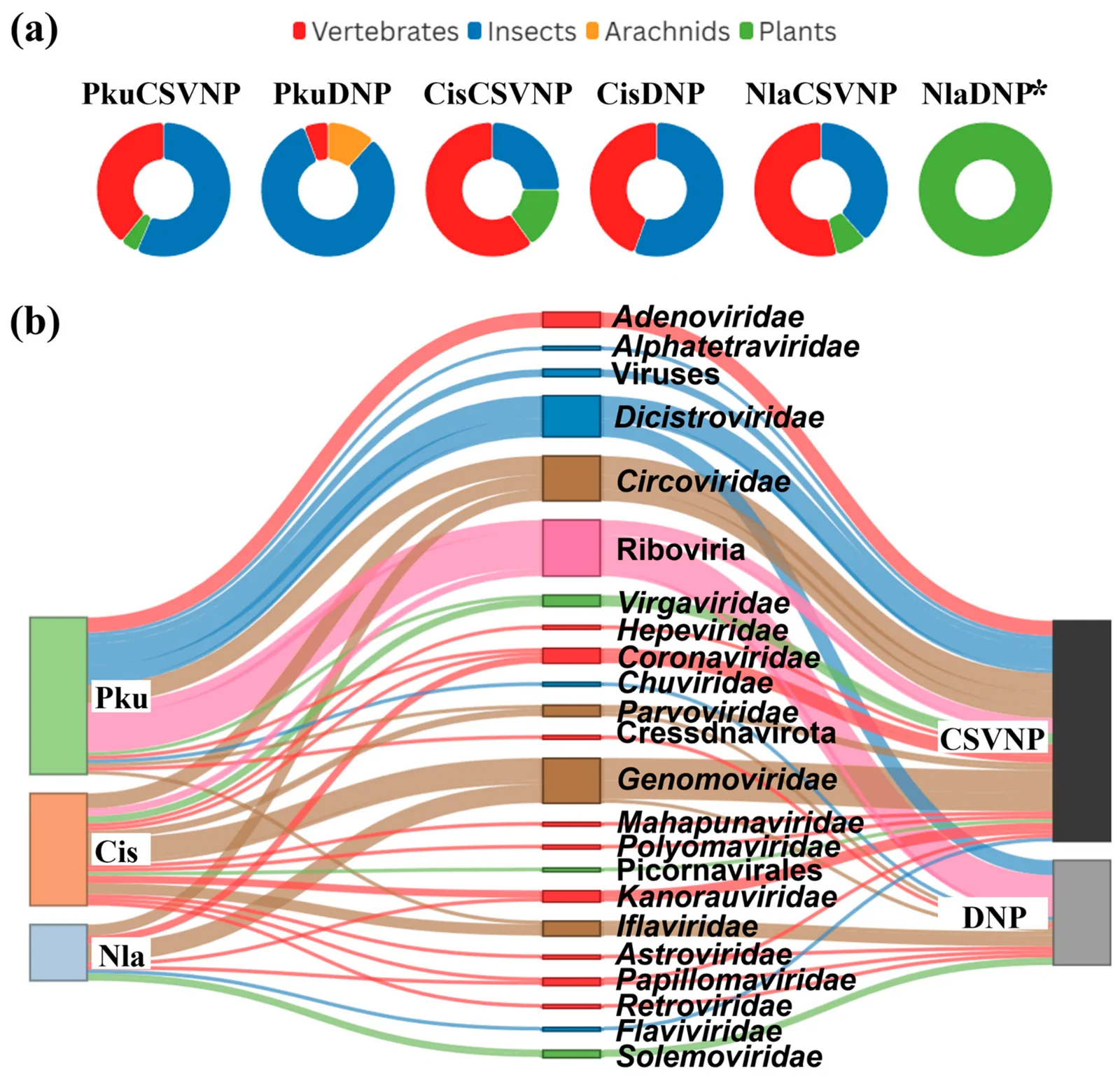
Composition (number of viral taxa) of the virome detected in samples from the different bat species across the studied environments: (**a**) Distribution of viral families according to host type; (**b**) graph of the 19 detected viral families, showing the connections between viral richness and relative abundance across samples from the three bat species (left) and the sampling environments, CSVNP and DNP (right). The width of the bars represents the relative abundance of viruses in bats and environments. Acronyms represent bat species and sampling environments as follows: **PkuCSVNP** (*P. kuhlii* from CSVNP); **PkuDNP** (*P. kuhlii* from DNP); **CisCSVNP** (*C. isabellinus* from CSVNP); **CisDNP** (*C. isabellinus* from DNP); **NlaCSVNP** (*N. lasiopterus* from CSVNP); and **NlaDNP** (*N. lasiopterus* from DNP). An asterisk (*) indicates that the corresponding dataset was represented by a single library.

The samples from *P. kuhlii* showed 37 viral species, and nine families were identified. Of these, 24 viral species (seven families) were found in CSVNP, and 17 species (three families) in DNP, with three *Dicistroviridae* species shared between the environments. The majority of detected viruses were insect-associated (Figure 5). Diversity analyses indicated higher viral richness and Shannon diversity in CSVNP than in DNP, as reflected by higher species richness and Hill number q = 0. However, diversity profiles converged at higher q-values, indicating that the samples from this species shared a similar set of dominant viral taxa in both environments. This pattern is consistent with the lack of significant differences in Shannon diversity (*p* = 0.5653 and 0.3062). It suggests that observed differences between habitats are primarily driven by richness and the contribution of rare viral taxa.

The samples from *C. isabellinus* harbored 31 viral species and 14 families, with 22 species (belonging to ten families) detected in CSVNP and nine species (belonging to six families) in DNP. The families *Genomoviridae* and *Parvoviridae* were found in both environments, and most of the viruses detected in this bat species were associated with vertebrates (Figure 5). Although species richness was markedly higher in CSVNP, this population showed lower evenness and Shannon diversity, indicating strong dominance by a subset of viral taxa. In contrast, DNP exhibited lower virus richness but higher evenness and Shannon diversity, reflecting a more equitable distribution of viral abundances. However, none of these differences appear to be significant (Table 3). As with the previous bat species, Hill diversity profiles revealed a steeper decline with increasing q in CSVNP. This further supports the hypothesis of a consistent richness–dominance trade-off between environments, which ultimately resulted in comparable Shannon diversity values with no significant differences between environments (*p* = 0.9012 and 0.4526).

Among the three species, *N. lasiopterus* exhibited the lowest viral diversity in its samples from CSVNP, the only environment in which the comparison was possible, with 14 viral species and seven families. In this environment, diversity metrics indicated a moderately structured viral community, with intermediate richness and evenness. Although Shannon diversity was relatively high, Hill’s diversity values indicate that viral abundance was concentrated in a limited number of dominant taxa. In DNP, only a single viral library of *N. lasiopterus* passed quality filtering, yielding one detected virus (Sobemovirus SOM). Therefore, diversity estimates for DNP were minimal and were not interpreted, as they likely reflect the limited number of informative libraries rather than true lower viral diversity.

Comparing environments, the samples from CSVNP harbored 51 viral species and 15 families, of which 45 species were exclusive to this environment. Notably, one of these viruses, exclusively detected in CSVNP, *Cyclovirus vazka*, was detected across all the bat species sampled. By contrast, the open land of DNP yielded 27 viral species across nine families, with 21 viruses unique to this environment (Figure 2). Although diversity indices did not differ significantly between environments, viral community composition differed markedly, reflecting differences in viral taxon composition rather than in dominance or evenness.

## 4. Discussion

Viruses are the most diverse and abundant biological entities on Earth and play fundamental ecological and evolutionary roles in shaping host evolution, population dynamics, and community structure [1,2]. Despite their importance, our understanding of viral diversity in animals remains uneven and strongly biased toward a limited number of host taxa [4]. At the same time, host–virus interactions do not occur in isolation but are embedded within ecological systems that are increasingly altered by human activities [14,15]. These changes in the landscape can alter the richness, abundance, and ecological interactions between wild species at all organizational levels, modifying contact rates, resource availability, and, eventually, community composition [34]. To better understand these complex dynamics, it is essential to examine host groups within their environmental contexts.

Within this ecological framework, bats represent a particularly informative model, as they are a highly diverse and widely distributed group of mammals, with distinctive evolutionary and ecological traits associated with high viral diversity [12,56]. In this study, we have analyzed and compared the viromes in samples of three phylogenetically related vespertilionid bats that differ markedly in body size, life history, and behavior. These species were sampled in two ecologically contrasting environments: a well-preserved Mediterranean forest dominated by pines and oaks (CSVNP) and a mosaic of agrosystems and marshlands modified by extensive livestock and agriculture (DNP). This environmental contrast provides an opportunity to explore how habitat conditions influence viral richness and composition across bat species with different ecological strategies.

Metagenomic sequencing revealed a complex and heterogeneous virome in the three bat species studied and in the two environments sampled. The majority of the 72 eukaryotic viral taxa detected in our samples have previously been reported in bat viromes from different regions of the world or associated with potential dietary sources [57,58,59,60,61,62,63,64,65,66,67]. Overall, in our samples, the RNA viruses predominated (59.72%), and viruses reported in insects, those linked to flying insects, were particularly abundant (45.83%), a result consistent with the predominantly insectivorous diet and aerial foraging strategy presented by the three studied bat species [68]. Viruses previously reported in vertebrates constituted the second most abundant group.

Most of the detected viral families have previously been reported in bats [57,58,59,60,61,69,70,71,72], except for *Chiunvirus melani* (*Kanorauviridae*) [73] and *Acamarivirus solis* (*Mahapunaviridae*) [74], which were documented in non-flying mammals. Our findings expand the known geographic distribution of several viral taxa and provide new records of their detection in bat samples. The relatively low proportion of viral sequences (6.94%) that correspond to plant-associated viruses is most likely due to ingestion by phytophagous insects. Collectively, these patterns highlight the significant impact of diet and environmental exposure on the composition of the bats’ virome.

Finally, although several of the detected viral families include zoonotic members, such as *Astroviridae*, *Coronaviridae*, *Hepeviridae*, and *Retroviridae*, none of the viral taxa identified in this study have been associated with human infection or disease.

### 4.1. Viral Diversity Across Samples from Different Bat Species

Viral diversity showed descriptive variation among samples from the three bat species analyzed. *P. kuhlii* exhibited the highest viral richness and diversity values, whereas *N. lasiopterus* showed the lowest values (at least in the well-preserved forest). However, statistical comparisons did not reveal significant differences among species. Therefore, the results do not provide statistical support for the hypothesis that viral diversity differs among bat species according to their ecological characteristics. Nevertheless, the observed patterns provide useful ecological context for interpreting differences in viral assemblage composition among the species.

The samples from *P. kuhlii* exhibited the highest viral diversity and were the only samples in which adenoviruses were detected. This observation is consistent with hypotheses linking increased viral diversity, and particularly adenovirus occurrence, to broad environmental tolerance in hosts [74]. Indeed, *P. kuhlii* occupies nearly all major European biogeographical regions (Alpine, Atlantic, Black Sea, Continental, Macaronesian, Mediterranean, Pannonian, and Steppic), and the species is clearly expanding northwards in recent times [75,76]. Moreover, it comprises multiple genetic lineages mainly across the Mediterranean basin. These two characteristics have been associated with increased viral diversity in bats [21,30,74,77]. Such ecological breadth probably enhances exposure to a wide array of viral sources.

The elevated viral richness observed in *P. kuhlii* is largely driven by viruses previously reported in insects, many of which correspond to taxa in its diet, including viruses associated with agricultural pests such as *Plautia stali* [40,68,78]. This bat is considered a dietary generalist, another characteristic associated with higher viral richness than expected [29]. Our results support the idea that feeding ecology plays an important role in determining the diversity of viral sequences detected in the bat samples.

In addition, the detection of viruses related to odonates (e.g., *Scarabeuvirus hubeiense*, *Cyclovirus vazka*, and *Hubei picorna-like virus 33*) and arachnids (*Hubei picorna-like virus 62* and *Wuhan spider virus 3*), prey groups not yet documented for this species, may reflect occasional consumption, secondary ingestion via infected insect prey, or incomplete dietary characterization. Together, this diversity of viruses highlights the complexity of viral acquisition pathways and suggests that bats may interact with a broader spectrum of arthropods than is currently reflected in dietary studies.

Notably, the high viral richness observed in *P. kuhlii*, the smallest of the three studied bats, contrasts with studies reporting a positive association between body mass and viral diversity, whereby larger bat species tend to harbor more viruses [8,77,79]. This discrepancy could reflect differences in the viral groups considered. Whereas previous studies focused primarily on bat-specific or vertebrate-related viruses, this study’s dataset, which is not a priori biased by the search, is dominated by insect-derived viruses, suggesting that the effect of body size on viral diversity may vary depending on the viral groups included in the analysis.

The viral profile observed in samples from *C. isabellinus* showed lower overall richness than that of samples from *P. kuhlii*, with a predominance of viruses previously reported in insects, and particularly in arthropod prey of this species [40,68,78]. Consistent with this interpretation, *Blattambidensovirus incertum1* (*Parvoviridae*) was detected in fecal samples. Although this virus was originally identified in tissue samples from the insectivorous bird *Parus major*, phylogenetic analyses place it within a lineage of densoviruses associated with insects, indicating that birds are unlikely to represent its natural host and that its original detection most likely reflected dietary acquisition from insect prey rather than a genuine host association [80,81].

Additional insect-related viruses expanded the spectrum of potential prey interactions. As with *P. kuhlii*, the detection of odonate-associated viruses (*Cyclovirus vazka*, *Gemycircularvirus odona1*, and *Gemycircularvirus odona2*) may indicate occasional predation on these taxa or incomplete representation of odonates in existing dietary surveys. Similarly, the presence of *Thaumetopoea pityocampa iflavirus 1*, associated with the pine processionary moth (*Thaumetopoea pityocampa*), likely reflects ingestion of this lepidopteran. Given that this moth is a major Mediterranean forest pest [82], its detection is ecologically relevant in the context of trophic interactions involving pest species.

The absence of rhabdoviruses in *C. isabellinus*, despite its recognized role as the primary reservoir of *European Bat Lyssavirus 1* (EBLV-1) in southern Iberia [83], is not unexpected, given the typically low prevalence of EBLV-1 reported in natural bat populations, even within established reservoir hosts [84].

Among the three studied species, *N. lasiopterus* (CSVNP) showed the lowest viral diversity (S and H1). This was an unexpected result, given that this bat is among the largest in Europe and shows well-documented long-distance movements and a broad foraging range [40], traits that could increase exposure to diverse environments and prey. Its large body size also contrasts with patterns reported in several studies, where larger bat species tend to harbor higher viral richness [8,74,77,79]. However, similar virus scarcity has been documented for the closely related *N. leisleri* and *N. noctula* in Switzerland, both also migratory species with long-range movements that nonetheless harbored fewer viruses than more sedentary bats [85]. Conversely, other migratory bats, such as *Pipistrellus nathusii* and *Vespertilio murinus*, exhibited higher viral richness than sedentary species [85]. These contrasting findings suggest that migration per se does not uniformly predict viral diversity and instead point to potential genus-level patterns within *Nyctalus*, possibly linked to other life-history traits specific to this group of bats.

Despite its lower overall richness, this species exhibited a relatively higher proportion of vertebrate-associated viruses, which aligns with patterns reported for other migratory European bats (*N. leisleri*, *N. noctula*, *P. nathusii*, and *V. murinus*) [85]. In addition, as observed in *P. kuhlii* and *C. isabellinus*, the viral composition in *N. lasiopterus* was strongly associated with diet [40,68,78]. Most insect-associated viruses detected in insectivorous bats are likely transient, as predator–prey viral transfer between phylogenetically distant hosts typically persists only briefly in the digestive tract before being eliminated [86]. This underscores the dynamic nature of prey-derived viromes and highlights the importance of distinguishing dietary from host-associated viral components in metagenomic studies.

### 4.2. Viral Diversity Across Different Environments

The two environments examined in this study differ markedly in their ecological characteristics and conservation status. CSVNP is characterized by a complex topography, high environmental heterogeneity, and exceptional biological richness [87,88], supporting the highest bat species richness reported within a single Iberian region (22 species) [41]. In contrast, DNP is a wetland ecosystem heavily influenced by agricultural intensification, hydrological alteration, loss of native vegetation, and comparatively low bat diversity (eight species) [13,14,44,45].

Consistent with these ecological differences, viral communities differed between environments, highlighting the influence of habitat context on bat hosts. CSVNP harbored greater viral richness than DNP at both the species and family levels; however, these differences were not reflected by Shannon diversity or Hill numbers, which were comparable between environments. This indicates that similar diversity values were not driven by the same viral taxa but instead resulted from comparable dominance structures, suggesting high compositional turnover without major changes in effective diversity.

The finding of this pattern is important in relation to our initial hypothesis that viral diversity would be higher in the environment-modified landscape. Our results do not support this prediction. Instead, the greater viral richness observed in CSVNP suggests that environmental modification does not necessarily increase the number of viral taxa detected in the bat samples. Rather, the sampled species-environment combinations are characterized primarily by differences in their viral taxonomic composition.

Such patterns are consistent with previous studies showing that the environment is a key driver of richness both at the species and family levels and in viral circulation of bats [29,59,89,90]. Conserved habitats generally support more diverse vertebrate communities, increasing opportunities for encounters among hosts and facilitating viral circulation through more complex ecological networks [36,38]. In addition, viruses capable of infecting multiple host species may persist more readily in species-rich communities, where increased host availability and interspecific interactions can promote transmission [36,38]. Accordingly, viruses previously reported in vertebrate hosts predominated in the samples from CSVNP, likely reflecting the greater diversity of potential vertebrate hosts in this environment.

In contrast, DNP exhibited lower viral richness and was characterized by a relatively greater representation of arthropod-associated viruses, arthropods typical of marshes, dunes, and riparian habitats. Because insectivorous bats acquire many dietary viruses through prey consumption, this pattern likely reflects differences in local arthropod assemblages, reinforcing the role of prey assemblages in shaping bat virome characteristics.

Although CSVNP contained a broader pool of viral taxa, viral abundance was concentrated in relatively few dominant species. Consequently, effective diversity remained similar between environments despite clear differences in richness and composition. This pattern suggests that biologically rich ecosystems may accumulate a larger repertoire of viruses without necessarily exhibiting a more even distribution of viral abundances.

Our findings agree with previous reports showing higher viral richness in conserved or less disturbed habitats than in anthropogenically modified systems, although patterns in diversity metrics are not clear bats [29,59]. In particular, studies using Hill numbers have reported that conserved environments often exhibit stronger dominance, whereas human-modified habitats tend to show greater evenness [59]. Likewise, vertebrate-associated viruses are generally more abundant in conserved ecosystems, while insect-associated viruses are proportionally more frequent in disturbed environments [29,59]. The greater representation of viruses previously reported in vertebrate hosts in the CSVNP environment is likely attributable to the greater abundance and diversity of vertebrate hosts, typically present in well-preserved habitats.

From an ecological perspective, pathogen transmission occurs within complex networks of interacting host species [36]. Consequently, changes in community composition and biodiversity are expected to alter viral circulation [37]. Theoretical models suggest that increasing biodiversity can enhance certain pathogen transmission under certain conditions. For example, transmission may increase when additional species function as competent hosts, increase contact rates, or enhance the persistence of infected individuals within the community [36]. The greater viral richness observed in CSVNP is consistent with these mechanisms, although host competence and transmission dynamics were not evaluated directly in this study.

These results suggest that the effects of habitat alteration on bat viromes depend, at least in part, on the ecological characteristics of individual viral families. Differences in host amplitude, transmission routes, environmental persistence, and host ecology may determine how each viral family responds to environmental change, rather than producing a uniform response across the virome. Similar family specific responses to habitat conservation and land-use change have been reported previously [90,91,92].

Overall, our results demonstrate that habitat context strongly influences viral richness and taxonomic composition in bat viromes while maintaining relatively stable patterns of dominance and effective diversity. Together, these findings highlight that environmental change primarily restructures which viral taxa are present rather than altering the overall distribution of viral abundances. Moreover, the prominence of insect-associated viruses across both environments emphasizes the importance of trophic interactions and local prey communities as key determinants of virome composition in insectivorous bats.

Taken together, the observed differences between environments should be interpreted in the context of the sampling design. Although the samples were initially collected from saliva or fecal material, the final analyses pooled both sample types for each bat species and environment. Consequently, the relative contribution of sample type to viral richness and composition cannot be evaluated independently from the environmental effects. Therefore, the differences observed between CSVNP and DNP should be interpreted as patterns associated with the sampled species–environment combinations rather than as evidence of a direct effect of environmental modification on viral diversity. This consideration is particularly relevant when interpreting the higher viral richness observed in CSVNP and the marked differences in viral taxonomic composition between environments.

### 4.3. Limitations and Perspectives

The interpretation of our findings should consider the methodological constraints inherent in pooled sampling designs. Pooling offers clear advantages, including reduced sequencing costs and a higher likelihood of detecting low-prevalence or rare viruses. However, it necessarily entails a loss of individual-level resolution, limiting our ability to estimate viral prevalence, detect coinfections, or assess inter-individual variability within bat populations.

In addition, differences in viral load among individuals may introduce compositional biases, as samples from hosts with high viral loads can disproportionately shape the pooled virome and potentially mask low-abundance taxa. While these limitations should be acknowledged, they do not diminish the utility of pooled metagenomic approaches for characterizing overall viral diversity and broad community structure. Rather, they highlight the need for caution when interpreting fine-scale ecological patterns or making epidemiological inferences based on pooled data. Finally, the use of high-quality filters can, as in this study, substantially reduce the number of libraries available for analysis and consequently limit the sample size for some species–environment comparisons. However, we considered that these filters were necessary to ensure data reliability and keep interpretations within reasonable limits.

An additional limitation concerns the taxonomic assignment of viral sequences. Sequences showing <95% nucleotide identity were conservatively classified as putative viral taxa; this may reflect divergence from available reference sequences or gaps in current viral databases and may limit the taxonomic resolution of these sequences. Beyond methodological considerations, our results emphasize the importance of integrating ecological, virological, and landscape-level perspectives to better understand viral assemblages in bat communities. A key challenge in wildlife virome studies remains distinguishing viral sequences derived from diet or environmental exposure from those reflecting active or persistent vertebrate infection.

Future research would benefit from expanded sampling across broader geographic and environmental gradients, combined with targeted molecular assays and, where feasible, virus isolation or other approaches to assess replication and infectivity, particularly for vertebrate-related viral groups. Such integrative frameworks will be essential for disentangling transient dietary signals from ecologically meaningful host–virus interactions and for interpreting virome diversity within a biodiversity-centered, ecosystem-based context.

## 5. Conclusions

This study represents the first comparative assessment of viromes in samples from bats across contrasting environments in the Iberian Peninsula. Despite the methodological limitations, uncertainty and complexity associated with the study of viromes in natural populations, our results point to the fact that viral diversity patterns are associated with host-specific traits and local ecological context.

Samples from CSVNP showed higher viral richness and a broader representation of viral families. However, higher viral diversity does not necessarily imply increased public health risk. In contrast, samples from DNP harbored fewer viral taxa overall but included viruses previously reported in taxa characteristic of wetland environments.

Despite these differences, effective diversity metrics were broadly comparable between environments, indicating compensatory dynamics between richness and dominance. Importantly, similar diversity values did not reflect the same viral taxa; rather, each environment supported a distinct viral assemblage with a comparable structural distribution of dominant and rare viruses.

At the species level, virome composition differed among samples from the three bat species. *P. kuhlii* showed the highest viral richness, likely associated with its ecological generalist and broad dietary spectrum, whereas *C. isabellinus* exhibited an intermediate profile dominated mainly by insect-associated viruses linked to prey consumption. In contrast, *N. lasiopterus* displayed comparatively low viral diversity despite its large body size and migratory behavior. Together, these findings suggest that viral diversity detected in bat samples is associated with interactions among habitat structure, host ecology, and trophic pathways.

## Figures and Tables

**Table 1:** Total number of bats captured (*n*), showing the study environments and species.

Environments	Species	*n*	Pools	Acronym
Sierras de Cazorla, Segura, and Las Villas Natural Park (CSVNP)	*Pipistrellus kuhlii*	17	8	PkuCSVNP
	*Cnephaeus isabellinus*	10	6	CisCSVNP
	*Nyctalus lasiopterus*	14	6	NlaCSVNP
Doñana National Park (DNP)	*Pipistrellus kuhlii*	15	6	PkuDNP
	*Cnephaeus isabellinus*	10	4	CisDNP
	*Nyctalus lasiopterus*	12	6	NlaDNP

**Table 2:** Number of reads per species and the studied environment, obtained through metagenomic analysis. Eukaryotic virus read count refers to those reads mapping to a reference genome and passing the quality control defined in Section 2.3. Acronyms represent the combinations of bat species and environments: PkuCSVNP (*P. kuhlii* from CSVNP); PkuDNP (*P. kuhlii* from DNP); CisCSVNP (*C. isabellinus* from CSVNP); CisDNP (*C. isabellinus* from DNP); NlaCSVNP (*N. lasiopterus* from CSVNP); and NlaDNP (*N. lasiopterus* from DNP). An asterisk (*) indicates that the corresponding dataset was represented by a single library.

Samples	Total Reads	Host Reads	Eukaryotic Virus Reads
PkuCSVNP	295,200,604	14,373,039	131,879
PkuDNP	109,012,724	9,450,828	118,701
CisCSVNP	999,673,482	79,416,117	6,721,148
CisDNP	547,137,756	56,211,029	18,230
NlaCSVNP	368,250,135	17,542,101	1,601,042
NlaDNP *	30,448,762	883,010	1931
∑	2,349,723,463	177,876,124	8,592,931

**Table 3:** Viral richness (S), evenness (E), and Shannon diversity (H′) for samples from each bat species in CSVNP and DNP, calculated at the viral species and viral family levels. (*P*) corresponds to tests for differences in Shannon diversity between sampling environments. Acronyms represent bat species and sampling environments as follows: **PkuCSVNP** (*P. kuhlii* from CSVNP); **PkuDNP** (*P. kuhlii* from DNP); **CisCSVNP** (*C. isabellinus* from CSVNP); **CisDNP** (*C. isabellinus* from DNP); **NlaCSVNP** (*N. lasiopterus* from CSVNP); and **NlaDNP** (*N. lasiopterus* from DNP).

Bat	CSVNP			DNP			
	S	E	H′	S	E	H′	*P*
Viral species							
**Pku**	24	0.6161	1.9582	17	0.5887	1.6679	0.5653
**Cis**	22	0.3462	1.0700	9	0.5194	1.1413	0.9012
**Nla**	14	0.5655	1.4924	1	1.0000	0.0000	0.4427
Viral family							
**Pku**	9	0.6343	1.3937	5	0.2860	0.4603	0.3062
**Cis**	12	0.1672	0.4154	7	0.5080	0.9885	0.4526
**Nla**	7	0.3500	0.6811	1	1.0000	0.0000	0.4936

## Data Availability

The original contributions presented in this study are included in the article/Supplementary Materials. Further inquiries can be directed to the corresponding authors.

## Supplementary Materials

The following supporting information can be downloaded at: https://www.mdpi.com/article/10.3390/microorganisms14092025/s1, Table S1. Pool composition and distribution across bat species and study environments. An as-terisk (*) indicates libraries that passed all predefined quality-control and viral se-quence criteria and were retained for downstream analyses; Table S2. Blast the result of contigs from Cazorla and Doñana samples.

## Abbreviations

BU-ISCIII	Bioinformatics Unit at the Health Institute Carlos III
CisCSVNP	*Cnephaeus isabellinus* from Sierras de Cazorla, Segura, and Las Villas Natural Park
CisDNP	*Cnephaeus isabellinus* from Doñana National Park
CSVNP	Sierras de Cazorla, Segura, and Las Villas Natural Park
^q^D	Hill number
DNA	Deoxyribonucleic acid
DNP	Doñana National Park
E	Evenness index
EBLV-1	European Bat Lyssavirus 1
EBD	Estación Biológica de Doñana
H′	Shannon diversity
MITECO	Ministerio para la Transición Ecológica y el Reto Demográfico
MEM	Minimum Essential Medium
MERS-CoV	Middle East Respiratory Syndrome Coronavirus
NGS	Next-generation sequencing
NlaCSVNP	*Nyctalus lasiopterus* from Sierras de Cazorla, Segura, and Las Villas Natural Park
NlaDNP	*Nyctalus lasiopterus* from the Doñana National Park
PCR	Polymerase chain reaction
PkuCSVNP	*Pipistrellus kuhlii* from Sierras de Cazorla, Segura, and Las Villas Natural Park
PkuDNP	*Pipistrellus kuhlii* from Doñana National Park
RNA	Ribonucleic acid
RPM	Reads per million
S	Richnes
SARS-CoV-1	Severe Acute Respiratory Syndrome Coronavirus 1
SARS-CoV-2	Severe Acute Respiratory Syndrome Coronavirus 2
SECIHTI	Secretaría de Ciencia, Humanidades, Tecnología e Innovación
